# *MeshAndCollect*: an automated multi-crystal data-collection workflow for synchrotron macromolecular crystallography beamlines

**DOI:** 10.1107/S1399004715017927

**Published:** 2015-10-31

**Authors:** Ulrich Zander, Gleb Bourenkov, Alexander N. Popov, Daniele de Sanctis, Olof Svensson, Andrew A. McCarthy, Ekaterina Round, Valentin Gordeliy, Christoph Mueller-Dieckmann, Gordon A. Leonard

**Affiliations:** a Structural Biology Group, European Synchrotron Radiation Facility, CS 40220, 38043 Grenoble, France; b European Molecular Biology Laboratory, Hamburg Outstation, Notkestrasse 85, 22607 Hamburg, Germany; c European Molecular Biology Laboratory, Grenoble Outstation, 71 Avenue des Martyrs, CS 90181, 38042 Grenoble, France; d Unit of Virus Host-Cell Interactions, Université Grenoble Alpes–EMBL–CNRS, 38042 Grenoble, France; e Université Grenoble Alpes, IBS, 38044 Grenoble, France; f CNRS, IBS, 38044 Grenoble, France; g CEA, IBS, 38044 Grenoble, France; h ICS-6: Molecular Biophysics, Institute of Complex Systems (ICS), Research Centre Juelich, 52425 Juelich, Germany; i Laboratory for Advanced Studies of Membrane Proteins, Moscow Institute of Physics and Technology, Dolgoprudniy 141700, Russian Federation

**Keywords:** macromolecular crystallography, synchrotron serial crystallography, multi-crystal data collection, hierarchical cluster analysis

## Abstract

Here, an automated procedure is described to identify the positions of many cryocooled crystals mounted on the same sample holder, to rapidly predict and rank their relative diffraction strengths and to collect partial X-ray diffraction data sets from as many of the crystals as desired. Subsequent hierarchical cluster analysis then allows the best combination of partial data sets, optimizing the quality of the final data set obtained. The results of applying the method developed to various systems and scenarios including the compilation of a complete data set from tiny crystals of the membrane protein bacterio­rhodopsin and the collection of data sets for successful structure determination using the single-wavelength anomalous dispersion technique are also presented.

## Abstract 2

The fully automated collection and merging of partial data sets from a series of cryocooled crystals of biological macromolecules contained on the same support is presented, as are the results of test experiments carried out on various systems.

## 1. Introduction   

Merging partial X-ray diffraction data sets from many crystals to produce a complete data set can be traced back to the very beginnings of macromolecular crystallography (MX). Indeed, in order to cope with the radiation damage observed at room temperature, the crystal structure solution of myoglobin required the merging of partial data sets, each comprising a single precession photograph, from 22 crystals per heavy-atom derivative (Kendrew *et al.*, 1960 ▸). However, with the introduction of cryogenic data-collection techniques (Hope, 1988 ▸) the effects of radiation damage could be limited dramatically. This generally allowed the collection of complete data sets from single crystals of biological macromolecules, even at beamlines at high-intensity third-generation synchrotron sources, and this soon became the norm.

The emergence of X-ray free-electron lasers (XFELs) saw multi-crystal data collection in MX revived and taken to its logical limits. Owing to the exceptionally bright nature of XFEL beams, experimenters adopted a ‘diffraction before destruction’ approach, dubbed serial femtosecond crystallo­graphy (SFX), streaming microcrystals through the X-ray beam and collecting still diffraction images where the crystal and X-ray laser pulse coincide (Chapman *et al.*, 2011 ▸). Complete data sets are then compiled by combining data from many thousands of still diffraction images. While SFX is likely to prove a watershed in MX, chiefly because the crystal structures determined using the technique should be largely free of radiation damage (Neutze *et al.*, 2000 ▸; Boutet *et al.*, 2012 ▸), the technique is not without its disadvantages. In particular, the amount of protein required in SFX experiments is currently rather high even for structure solution based on molecular-replacement techniques (*i.e.* only native data sets are collected). Moreover, although software for SFX data analysis is rapidly developing (Sauter *et al.*, 2013 ▸; White *et al.*, 2012 ▸, 2013 ▸; Barty *et al.*, 2014 ▸; Sawaya *et al.*, 2014 ▸), as the diffraction images collected in such experiments contain predominantly partially recorded reflections measured from crystals of different sizes with laser pulses of different spectral content, estimation of the intensity (and its standard deviation) of any given reflection is problematical and data-processing methods will have to evolve significantly if the quality of SFX-collected data is to approach that currently available in ‘traditional’ MX experiments.

Inspired by the success of SFX, experimenters at synchrotron MX beamlines have used similar paradigms (bright X-ray beams, fast read-out detectors, small crystals, single-exposure experiments) to develop synchrotron serial crystallography (SSX), showing that it is possible to compile useful data sets from hundreds or thousands of crystals introduced into the synchrotron beam either *via* jets (Nogly *et al.*, 2015 ▸), liquid streams in glass capillaries (Stellato *et al.*, 2014 ▸), free-standing high-viscosity micro-streams (Botha *et al.*, 2015 ▸), sandwiched between two silicon nitride (Si_3_N_4_; Coquelle *et al.*, 2015 ▸) or cyclic olefin copolymer (COC; Huang *et al.*, 2015 ▸) wafers that are translated through the X-ray beam, or contained on a cryocooled sample holder (Gati *et al.*, 2014 ▸). In the latter case the whole sample mount is continuously rastered through the X-ray beam, being rotated at the same time (de Sanctis *et al.*, 2012 ▸), and diffraction images are recorded on the fly at set time intervals. As for crystals introduced into the X-ray beam in liquid streams or on silicon nitride wafers, the large majority of diffraction images collected contain no useful information. However, that fact that the sample is also rotated while being rastered means that where the crystal and the X-ray beam coincide some diffraction images could contain fully recorded reflections, thus rendering the processing and scaling of diffraction images using standard software packages relatively straightforward and improving the overall data quality. Moreover, for crystals larger than the X-ray beam, diffraction images can be grouped into those originating from the same crystal, thus also facilitating data processing and improving the resulting data quality (Gati *et al.*, 2014 ▸).

While for the same crystal volume and X-ray beam size the resolution obtainable in SSX experiments is likely to always be lower than that in SFX, SSX will become an important technique in MX. In particular, initial crystals of many systems are often small and SSX provides a means to study them without the need for the often time-consuming and cumbersome optimization of crystal size and/or quality. Indeed, when combined with the extremely bright X-rays beams that will be available at future low-emittance fourth-generation storage rings (see, for example, http://www.esrf.fr/Apache_files/Upgrade/ESRF-orange-book.pdf), such experiments may well become the norm. However, even when rastering samples contained on a cryocooled sample holder through the X-ray beam, SSX often suffers, as does SFX, from the fact that no attempt is made to synchronize the intersection of the X-ray beam and crystal during the experiment. Moreover, as the SFX ‘diffraction before destruction’ principle currently does not apply in SSX experiments on cryocooled samples, the amount of diffraction data collected from any given crystal is far from optimized.

**Figure 1:**
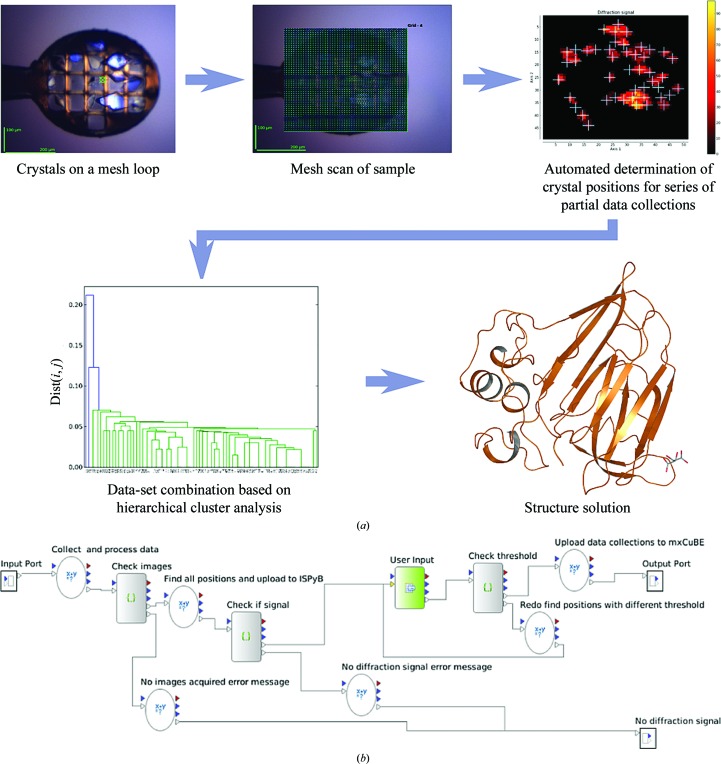
The *MeshAndCollect* workflow for a multicrystal data-collection method. (*a*) A mesh scan is performed on the sample. The resulting images are automatically inspected for protein diffraction and scored according to diffraction strength. A heat map is generated that represents the diffraction intensity, where the positions for partial data collections are marked. After the user has selected the settings for the partial data collections, the *MxCuBE*2 data-collection queue is automatically filled and all partial data sets are collected. Once the partial data sets have been automatically processed, HCA can then be used to choose which data sets to merge to produce a final data set for structure solution. (*b*) Flow diagram of the *MeshAndCollect* workflow used.

Recent developments based on either the optical (Huang *et al.*, 2015 ▸) or diffraction-based (Soares *et al.*, 2014 ▸) pre-interrogation of multi-crystal sample holders have ensured the synchronization of X-ray beam and crystals in SSX protocols and have enormously reduced the amount of sample required for a successful experiment. In a further step towards the optimal collection of diffraction data in SSX experiments from samples which can sustain the collection of many X-ray diffraction images before significant radiation damage occurs, we have developed an automatic procedure (Fig. 1 ▸). Here, the positions of many randomly oriented (micro)crystals contained in a single cryocooled sample holder are determined using an X-ray-based two-dimensional scan, the diffraction strength of each crystal found is automatically ranked and partial data sets from each crystal are collected and processed online. Subsequent manual hierarchical cluster analysis (HCA; Giordano *et al.*, 2012 ▸) is then used to decide the most correlated partial data sets to merge to produce the best quality data set for use in downstream analysis and structure solution. The protocol developed can in principle be applied to crystals mounted in almost any type of currently available mounting platform (*i.e.* nylon loops, micro-meshes, Si_3_N_4_ or COC wafers *etc.*) and is applicable not only to multi-crystal data collection but additionally automates multi-position data collection from large crystals when exploiting mini-focus or micro-focus X-ray beams.

As proof of the general usefulness of the protocol developed, we present the results of applying this method to various systems and scenarios. These include the compilation of a complete data set from microcrystals of the membrane protein bacteriorhodopsin, the collection and merging of partial data sets collected from different positions of larger crystals and the collection of data sets for use in structure determination using single-wavelength anomalous dispersion techniques.

## 2. Methods   

In the experiments described here, the best results were obtained from crystals mounted in a flat sample holder (*i.e.* MiTeGen MicroMeshes; MiTeGen, USA; Fig. 1 ▸ *a*), avoiding stacking of crystals and an excess of surrounding mother liquor, before either flash-cooling in liquid nitrogen or directly on the beamline. When mounted on a goniometer, the plane of the sample holder should be perpendicular to the direction of the X-ray beam. This ensures that any crystal brought into the X-ray beam will remain illuminated over a relatively small rotation range (±5° in the experiments described here^1^). To make this adjustment, we usually exploit the mini-kappa goniometers (Brockhauser *et al.*, 2013 ▸) installed on most of the MX beamlines at the ESRF. The *MeshAndCollect* protocol (Fig. 1 ▸ *b*) is implemented in a customized Passerelle-EDM workflow engine (http://isencia.be/passerelle-edm-en) called the *Beamline Expert System* that is based on previous developments (Brockhauser *et al.*, 2012 ▸) and is embedded in the *MXCuBE*2 beamline-control graphical user interface (Gabadinho *et al.*, 2010 ▸; de Sanctis & Leonard, 2014 ▸). Once the workflow has been launched the user defines the size of the X-ray beam to be used. Ideally, this should correspond to, or be smaller than, the minimum dimension of the crystals contained in the sample holder. The area over which the initial two-dimensional mesh scan is performed (Fig. 1 ▸ *a*) is drawn by the user, with the dimensions of the grid and the X-ray beam size defining the number of points in the mesh scan. Diffraction images collected at each of these points are analysed on the fly for protein diffraction using the software *DOZOR* (§[Sec sec2.1]2.1). The user receives a heat map (Fig. 1 ▸), also stored in the ISPyB database (Delagenière *et al.*, 2011 ▸), showing the grid points at which diffraction has been observed. The user then has the possibility of adjusting the contrast level to include or exclude points for subsequent data collection. In the last experimental step partial data sets (±5° total rotation range, 100 images per partial data set) are collected sequentially at each grid point with a *DOZOR* score above the threshold. Each partial data set is automatically processed using the *GrenADes* pipeline (Monaco *et al.*, 2013 ▸) based on *XDS* (Kabsch, 2010 ▸) running in parallel with the data collection. Partial data sets that have been successfully processed are then scaled together using *XSCALE* (Kabsch, 2010 ▸). The resulting CC_*I*_(*i*, *j*) values calculated for the common unique intensities of each pair of data sets are used in a HCA protocol (Giordano *et al.*, 2012 ▸) to produce a dendrogram (Fig. 1 ▸). This is then used to decide which partial data sets to combine to produce, using the *CCP*4 programs *POINTLESS* and *AIMLESS* (Evans & Murshudov, 2013 ▸), the final data set for structure solution and refinement (Fig. 1 ▸). A feature of *POINTLESS* is that it uses the first partial data set provided as input as a reference data set. This avoids, where it might have been possible during automatic data processing, indexing ambiguities between partial data sets, with the result that the merged data set obtained is not artifactually merohedrally twinned (for a discussion of this, see Brehm & Diederichs, 2014 ▸). Any twinning then detected (*i.e.* using the ‘*H*-test’; Yeates, 1997 ▸) in the final merged data set, although an average over all crystals included, is likely to be real, facilitating determination of the true space group for use with the correct twinning fraction (if appropriate) in subsequent structure solution and refinement. 

### 2.1. *DOZOR*   

One of the core features of the protocol described here is the ability to automatically recognize and rank the series of single diffraction patterns collected during the low-dose mesh scan of the sample holder. This is carried out using the program *DOZOR*. As the algorithm used will be illustrated in more detail elsewhere, it will be only briefly described here.

In a first step, *DOZOR* determines the distribution of background intensity on a diffraction image as a function of the diffraction vector length *h*. This is accomplished by the iterative summation of pixel intensities and the sequential rejection of outliers. After azimuthal averaging this produces the one-dimensional background function 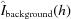. This function should be smooth: any sharp peaks are an indication of ice rings or salt diffraction, and such areas are not used in further calculations.

In the case of diffraction from a crystal of a biological macromolecule, the function
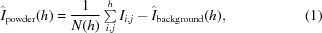
where *N*(*h*) is the number of detector pixels and *I*_*i*,*j*_ is the intensity in any pixel which belongs to the resolution shell, *h*), will give the estimate of the mean intensity of Bragg spots as a function of resolution and will represent the well known Wilson plot, which for any protein crystal can be modelled using 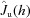, the unique pattern of average squared structure-factor magnitudes (Bourenkov & Popov, 2006 ▸). *DOZOR* approximates the experimental data by applying an isotropic Debye–Waller factor to the standard protein Wilson plot model,
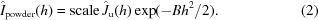



The quality of the resulting fit is evaluated *via* the correlation coefficient between the left and right parts of (2), CC_powder_. The program also identifies individual Bragg spots and makes a few simple geometrical checks which additionally validate the presence of diffraction from macromolecular crystals and allow the rejection of ice or salt contamination. Finally, a score of diffraction strength is estimated as the total averaged diffraction intensity multiplied by CC_powder_, where *V*(*h*) is the reciprocal volume of the resolution shell, 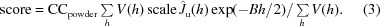
In the case where *DOZOR* cannot find any Bragg spots, the score is determined as zero.

## 3. Results   

### 3.1. Bacteriorhodopsin   

**Figure 2:**
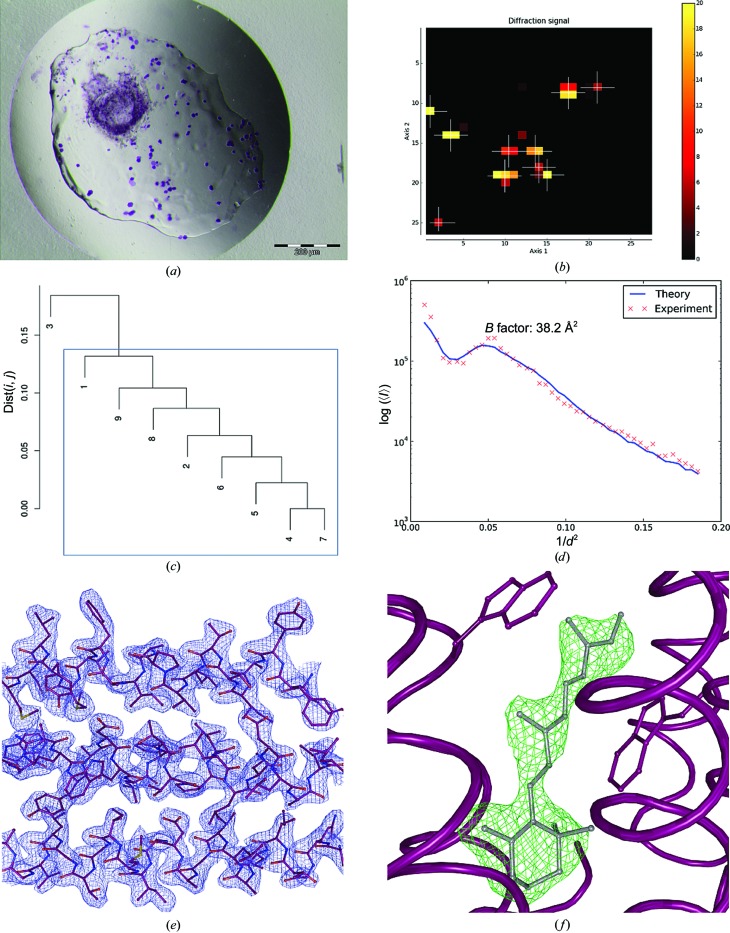
Multi-crystal data collection and structure solution from larger crystals of bacteriorhodopsin. (*a*) Crystals of bacteriorhodopsin obtained from crystallization in lipidic mesophase (Borshchevskiy *et al.*, 2011 ▸); the average crystal size is ∼20 × 20 × 5 µm. (*b*) Heat map after initial mesh scan of the sample holder. The colours from dark red to yellow represent the intensity of the detected diffraction signal at the respective position; the white crosses mark the positions that have been used for collection of partial data sets. In all heat plots shown the *x* axis represents the grid points along the horizontal translation of the sample holder and the *y* axis the vertical grid points. For both, the unit is the beam size. (*c*) Dendrogram based on HCA of CC_*I*_(*i*, *j*) values produced by *XSCALE*. The blue rectangle shows the partial data sets merged to produce the final data set. (*d*) Wilson plot derived from the final data set using *BEST* (Bourenkov & Popov, 2006 ▸). (*e*) Detail of the final 2*mF*_obs_ − *DF*_calc_, α_calc_ electron-density map (contoured at 1.5 × r.m.s.) obtained, with the refined structure shown in ball-and-stick representation. (*f*) OMIT difference density (*mF*_obs_ − *DF*_calc_, α_calc_) map at the end of the refinement procedure (contoured at 2.5 × r.m.s.) for a retinal molecule (ball-and-stick representation).

**Figure 3:**
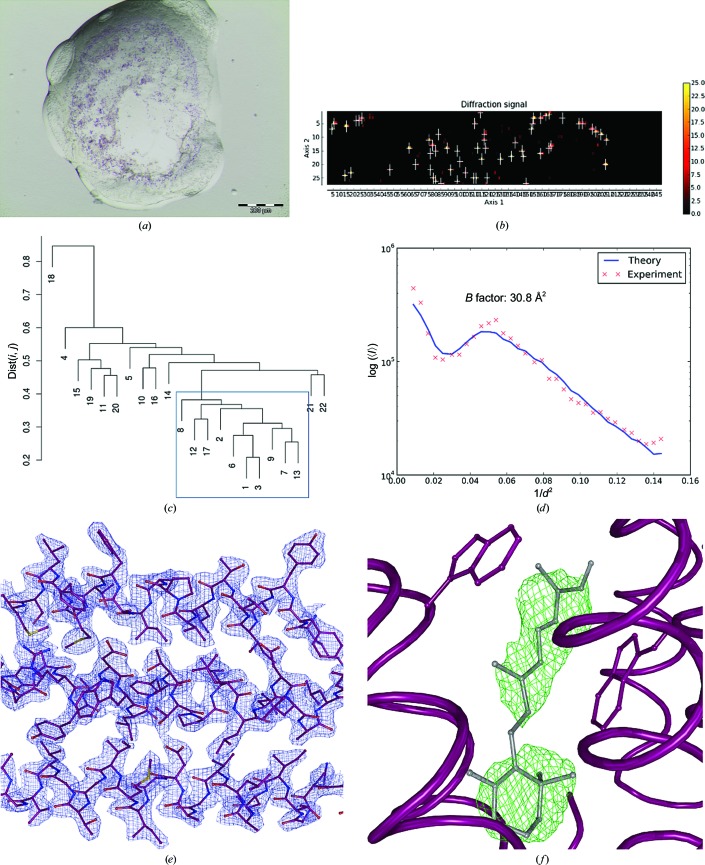
Multi-crystal data collection and structure solution from microcrystals of bacteriorhodopsin. (*a*) Microcrystals of bacteriorhodopsin obtained from crystallization in lipidic mesophase; average crystal size ∼5 × 5 × 2 µm. (*b*) Heat map after initial mesh scan of the sample holder. (*c*) Dendrogram based on HCA of CC_*I*_(*i*, *j*) values produced by *XSCALE*. (*d*) Wilson plot from the final data set derived using *BEST*. (*e*) Detail of the final 2*mF*_obs_ − *DF*_calc_, α_calc_ electron-density map (contoured at 1.5 × r.m.s.), with the refined structure shown in ball-and-stick representation. (*f*) OMIT difference density (*mF*_obs_ − *DF*_calc_, α_calc_) map at the end of the refinement procedure (contoured at 2.0 × r.m.s.) for a retinal molecule (ball-and-stick representation).

Crystals of bacteriorhodopsin (BR) were prepared as described previously (Gordeliy *et al.*, 2003 ▸). In this study, two batches of bacteriorhodopsin crystals were used: BR1 (Fig. 2 ▸ *a*), with dimensions of ∼20 × 20 × 5 µm, and BR2 (Fig. 3 ▸ *a*), with dimensions of ∼5 × 5 × 2 µm. Diffraction data (Table 1 ▸) were collected on ESRF beamline ID29 (de Sanctis *et al.*, 2012 ▸) using a PILATUS3 6M pixel detector (Dectris, Baden, Switzerland).

For BR1 the initial mesh scan was carried out using a Gaussian X-ray beam of 20 µm in diameter with a flux of 3 × 10^11^ photons s^−1^. The resulting heat map (Fig. 2 ▸ *b*) revealed ten well diffracting positions from which partial data sets were collected. All partial data sets could be automatically processed and, after HCA (Fig. 2 ▸ *c*), nine were chosen for scaling and merging to produce a final data set to *d*_min_ = 2.3 Å (Table 1 ▸; Wilson plot shown in Fig. 2 ▸ *d*).

For BR2, the initial mesh scan (X-ray beam of 10 µm in diameter with a flux of 1.5 × 10^11^ photons s^−1^) produced a heat map (Fig. 3 ▸ *b*) showing 59 diffracting positions in the sample holder from which partial data sets were collected. 38 partial data sets could be automatically processed and, after HCA (Fig. 3 ▸ *c*), ten were merged to produce a final data set to *d*_min_ = 2.6 Å; Table 1 ▸; Wilson plot shown in Fig. 3 ▸ *d*).

For both BR1 (twinning fraction 0.06) and BR2 (twinning fraction 0.39) structure solution was carried out by molecular replacement using *MOLREP* (Vagin & Teplyakov, 2010 ▸) with PDB entry 3ns0 (Borshchevskiy *et al.*, 2011 ▸) stripped of water molecules and ligands as a search model. Structure refinement (Table 2 ▸) was carried out using the twinning refinement option in *REFMAC*5 (Murshudov *et al.*, 2011 ▸) interspersed with rounds of manual rebuilding in *Coot* (Emsley *et al.*, 2010 ▸). In both crystal structures assignment of the retinal cofactor was possible from the interpretation of both electron-density and difference density maps and is well defined both in the final 2*mF*_obs_ − *DF*_calc_ electron density and in OMIT difference density maps (Figs. 2 ▸ *e*, 2 ▸ *f*, 3 ▸ *e* and 3 ▸ *f*).

### 3.2. Thaumatin   

**Figure 4:**
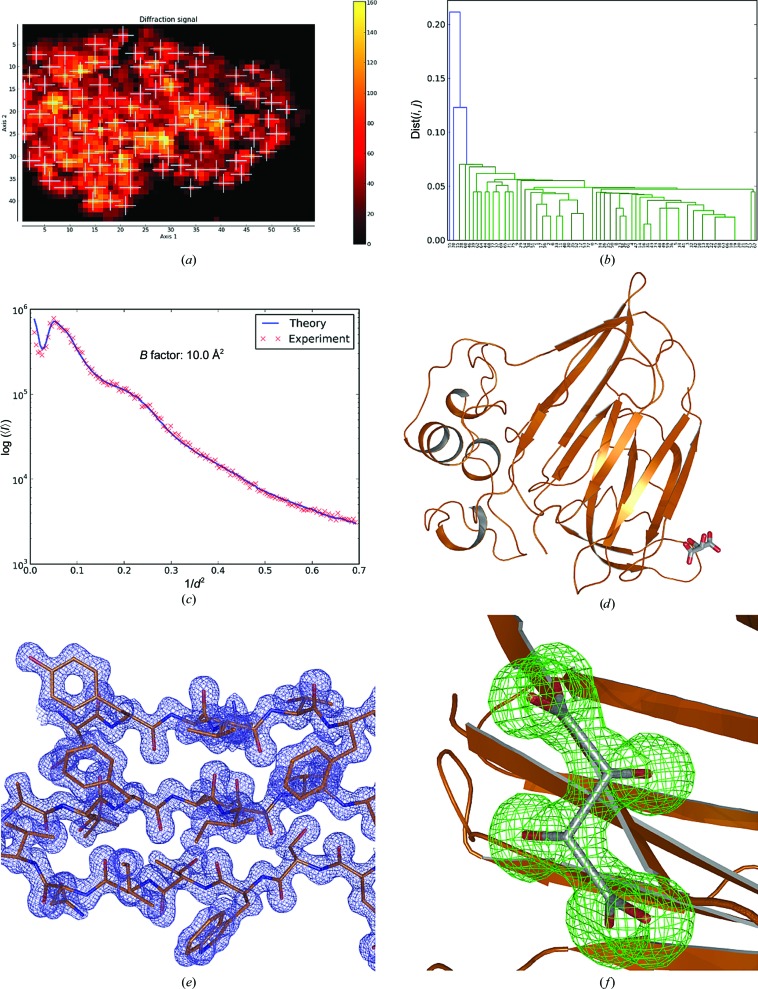
Multi-crystal data collection and structure solution from crystals of thaumatin. (*a*) Heat map after initial mesh scan of the sample holder. (*b*) Dendrogram based on HCA of CC_*I*_(*i*, *j*) values produced by *XSCALE*. (*c*) Wilson plot from the final data set derived using *BEST*. (*d*) A ribbon diagram of the refined crystal structure of thaumatin produced (tartrate molecule in stick representation). (*e*) Detail of the final 2*mF*_obs_ − *DF*_calc_, α_calc_ electron-density map (contoured at 1.5 × r.m.s.), with the refined structure shown in ball-and-stick representation. (*f*) Difference density (*mF*_obs_ − *DF*_calc_, α_calc_) for a tartrate molecule after structure refinement (OMIT map). The difference density is shown at a contour level of 3 × r.m.s.

Thaumatin (Sigma–Aldrich catalogue No. T7638) was dissolved in double-distilled water to a concentration of 20 mg ml^−1^. Crystals of approximate dimensions 40 × 40 × 60 µm were obtained in 2 µl (1:1 ratio) hanging drops using 0.1 *M* HEPES pH 7.5, 0.7 *M* potassium/sodium tartrate, 20% glycerol as a reservoir. Crystals were mounted as described in §[Sec sec2]2 without further cryoprotection. Data were collected on ESRF beamline ID29. The initial mesh scan was performed with an X-ray beam of 10 µm in diameter with a flux of 8.7 × 10^11^ photons s^−1^. From the resulting heat map (Fig. 4 ▸ *a*), 100 well diffracting points were chosen for the collection of partial data sets, of which 78 could be automatically integrated. After HCA (Fig. 4 ▸ *b*) 74 were merged to produce a final data set to *d*_min_ = 1.2 Å (Table 1 ▸; Wilson plot shown in Fig. 4 ▸ *c*).

Structure solution was carried out by molecular replacement using *MOLREP* with PDB entry 4axu (Cipriani *et al.*, 2012 ▸) stripped of water molecules and ligands as a search model. Structure refinement (Table 2 ▸, Fig. 4 ▸ *d*), during which analysis of difference electron-density maps clearly allowed the assignment of tartrate (one molecule; Figs. 4 ▸ *e* and 4 ▸ *f*) and glycerol (one molecule) moieties bound to the protein, was carried out in *REFMAC*5 alternated with manual rebuilding in *Coot*.

### 3.3. Monoclinic lysozyme   

**Figure 5:**
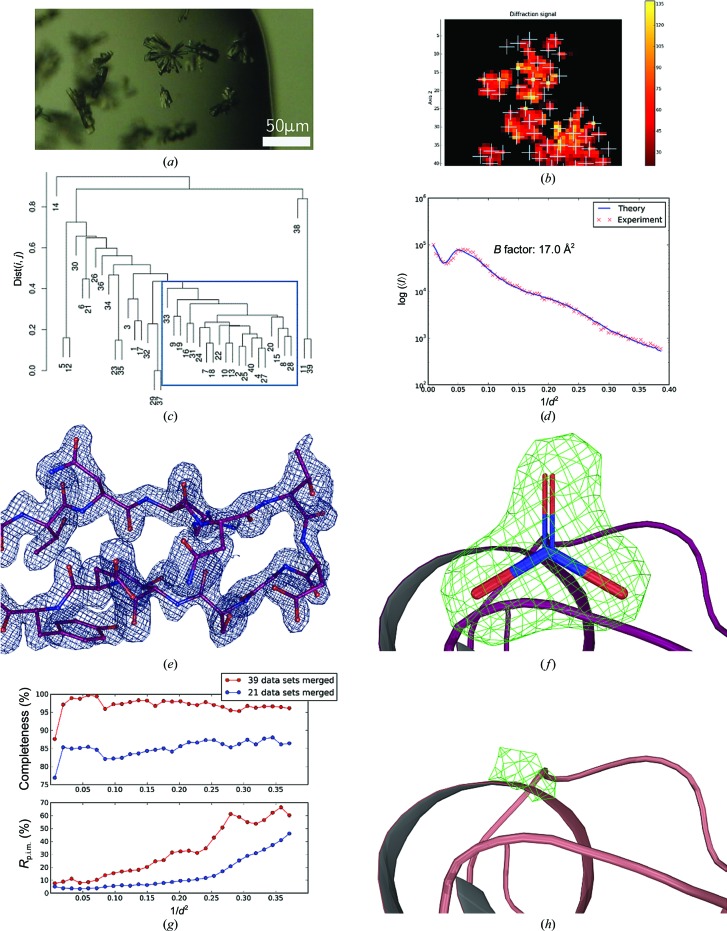
Multi-crystal data collection and structure solution from crystals of monoclinic lysozyme. (*a*) The ‘flowers’ of monoclinic lysozyme crystals produced by the crystallization procedure. (*b*) The heat map after an initial mesh scan of the sample used in the workflow described here. (*c*) Dendrogram based on HCA of CC_*I*_(*i*, *j*) values produced by *XSCALE*. (*d*) Wilson plot from the final data set derived using *BEST*. (*e*) Detail of the 2*mF*_obs_ − *DF*_calc_, α_calc_ electron-density map at the end of the refinement procedure (contoured at 1 × r.m.s; amino-acid residues shown in ball-and-stick representation). (*f*) Difference density (*mF*_obs_ − *DF*_calc_, α_calc_) for a nitrate molecule at the end of the structure-refinement procedure (OMIT map). The difference density is shown at a contour level of 3 × r.m.s. (*g*) Plots showing comparisons of the completeness (top panel) and quality of data sets obtained following either the HCA-directed merging of data sets (21 data sets merged, blue) or the ‘blind’ merging of 39 of the 40 data sets collected. (*h*) Difference density (*mF*_obs_ − *DF*_calc_, α_calc_) for a nitrate molecule at the end of the structure-refinement procedure based on the data set obtained by merging 39 of the 40 data sets collected. The difference density is shown at a contour level of 2.5 × r.m.s.

Lysozyme (Roche Applied Science, catalogue No. 10837059001) was dissolved in double-distilled water to a concentration of 40 mg ml^−1^. ‘Flowers’ of monoclinic (space group *P*2_1_) lysozyme crystals (Fig. 5 ▸ *a*), with each petal ∼80 µm in the largest dimension, were then obtained from 2 µl (1:1 ratio) hanging drops using 0.6 *M* NaNO_3_ as the precipitant/reservoir. Prior to mounting, 1 µm 75% glycerol was added to the crystallization drop for cryoprotection. Diffraction data were collected on ESRF beamline ID23-1 (Nurizzo *et al.*, 2006 ▸) using an X-ray beam of 10 µm in diameter with a flux of 3.5 × 10^10^ photons s^−1^. The initial mesh scan produced a heat map (Fig. 5 ▸ *b*) which was used as the basis for the collection of 54 partial data sets, of which 40 could be automatically processed. After HCA (Fig. 5 ▸ *c*) 21 partial data sets were merged to produce a final data set to *d*_min_ = 1.6 Å (Table 1 ▸; Wilson plot shown in Fig. 5 ▸ *d*). Structure solution and refinement (Table 2 ▸, Fig. 5 ▸ *d*) were then carried out as described above for thaumatin (using PDB entry 4axt stripped of water molecules and ligands as the search model for molecular replacement; Cipriani *et al.*, 2012 ▸), during which analysis of electron-density and difference electron density maps allowed the assignment of a nitrate (NO_3_^−^) ion bound to one of the lysozyme molecules in the asymmetric unit (Fig. 5 ▸ *f*).

### 3.4. Thermolysin   

**Figure 6:**
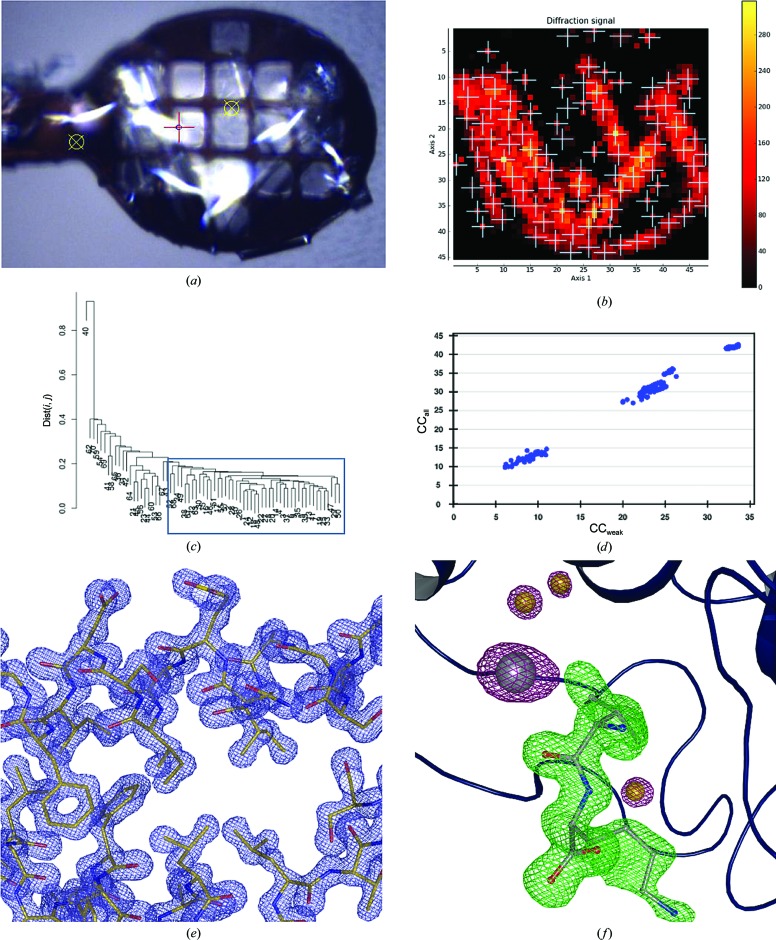
Multi-crystal data collection and SAD structure solution from crystals of thermolysin. (*a*) The sample holder mounted on ID23-1 immediately before launching the *MeshAndCollect* workflow. (*b*) The heat map after an initial mesh scan of the sample clearly shows the size and disposition of the crystals contained on the sample holder. (*c*) Dendrogram based on HCA of CC_*I*_(*i*, *j*) values produced by *XSCALE*. (*d*) A plot of CC_all_ *versus* CC_weak_ from *SHELXD*/*HKL*2*MAP* for trial substructures, clearly indicating successful substructure solution. (*e*) Detail of the final 2*mF*_obs_ − *DF*_calc_, α_calc_ electron-density map at the end of the refinement procedure (contoured at 1.5 × r.m.s; amino-acid residues in ball-and-stick representation). (*f*) Detail showing both anomalous difference map (Δ*F*_ano_, α_calc_ + 90°) peaks (purple chicken wire) around the catalytic Zn^2+^ ion (grey sphere) and three Ca^2+^ ions (yellow spheres) and OMIT difference density (*mF*_obs_ − *DF*_calc_, α_calc_, green chicken wire) in the region of a Val-Lys dipeptide found bound in the active site. The OMIT difference density is contoured at 3 × r.m.s. and the anomalous difference density at 4.5 × r.m.s.

*Bacillus thermoproteolyticus* thermolysin (Sigma–Aldrich catalogue No. T0331) was dissolved to 100 mg ml^−1^ in 45% DMSO, 0.05 *M* MES pH 6.0. The reservoir contained 35% saturated ammonium sulfate, whereas the drops were composed of the protein solution and a solution consisting of 0.05 *M* MES pH 6.0, 1 *M* NaCl, 45% DMSO in a 1:1 ratio. Rod-shaped crystals of between 40 × 40 × 150 and 40 × 40 × 300 µm in size were quick-soaked in 6 *M* trimethylamine *N*-oxide (TMAO; Mueller-Dieckmann *et al.*, 2011 ▸) for cryoprotection before mounting on a sample support (Fig. 6 ▸). Diffraction data were collected using an X-ray beam of 10 µm in diameter with a flux of 4.0 × 10^10^ photons s^−1^ at the peak of the Zn *K* absorption edge (λ = 1.256 Å) on beamline ID23-1 of the ESRF. The initial mesh scan produced a heat map (Fig. 6 ▸ *a*) which was used as a basis for the collection of 96 partial data sets, 77 of which were automatically processed and 49 were manually merged after HCA analysis to produce a final data set to *d*_min_ = 1.37 Å (Table 1 ▸, Figs. 6 ▸ *b* and 6 ▸ *c*). Structure solution (Fig. 6 ▸ *d*) was carried out using the SAD method (Dauter *et al.*, 2002 ▸) using the *SHELXC*/*D*/*E* pipeline (Sheldrick, 2008 ▸) as implemented in *HKL*2*MAP* (Pape & Schneider, 2004 ▸), with the initial *de novo*-obtained model of the crystal structure refined (Table 2 ▸, Fig. 6 ▸ *e*) using iterative rounds of *REFMAC*5 and manual rebuilding in *Coot*.

Our experiments with crystals of thermolysin reveal other features of the developed pipeline. In particular, when, as was the case here, the sample holder contains a series of crystals much larger than the X-ray beam (Fig. 6 ▸ *a*) multi-crystal/multi-position data collection is also automated. Indeed, for crystals that are larger than the X-ray beam the rapid online analysis and ranking of diffraction characteristics using *DOZOR* (§[Sec sec2.1]2.1) provides diffraction cartographs (Bowler *et al.*, 2010 ▸) of the crystals contained on the sample mount (Fig. 6 ▸ *b*). The workflow thus ensures that partial data sets are collected from only well diffracting areas of any given crystal.

### 3.5. MAEL domain of *Bombyx mori* Maelstrom   

**Figure 7:**
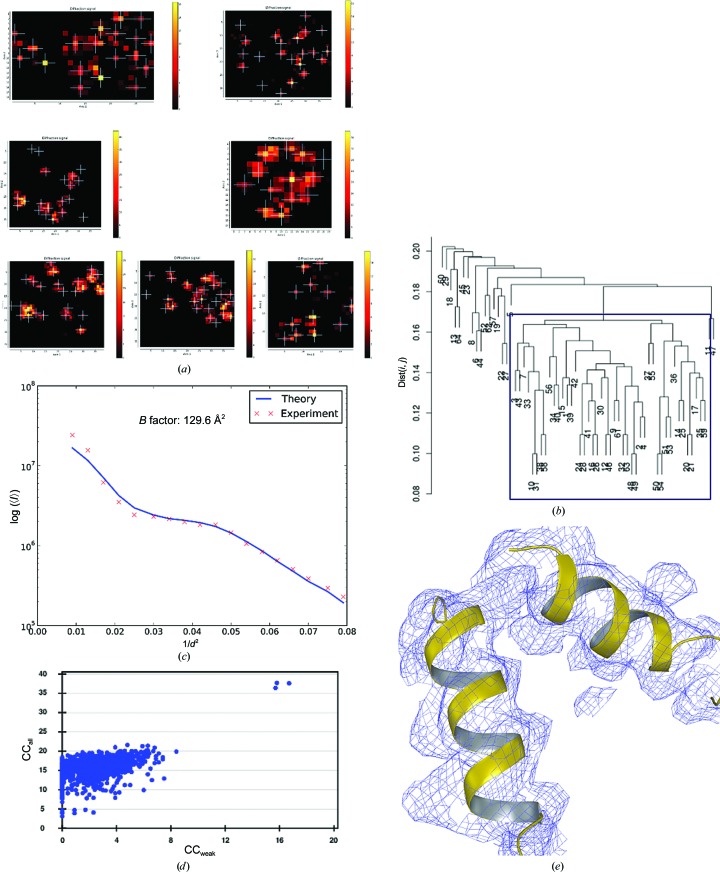
Multi-crystal data collection and SAD structure solution of Maelstrom. (*a*) Heat maps from initial mesh scans of the six sample holders analysed. (*b*) Dendrogram based on HCA of CC_*I*_(*i*, *j*) values produced by *XSCALE*. (*c*) Wilson plot from the final data set derived using *BEST*. (*d*) A plot of CC_all_ *versus* CC_weak_ from *SHELXD*/*HKL*2*MAP* for trial substructures, clearly indicating successful substructure solution. (*e*) Representative part of the 2*mF*_obs_ − *DF*_calc_, α_calc_ electron-density map after initial model building and refinement, with two α-helices shown in ribbon representation.

Diffraction data from crystals of the selenomethionyl derivate of the MAEL domain of *B. mori* Maelstrom (for crystallization conditions, see Chen *et al.*, 2015 ▸) were collected using an X-ray beam of 10 µm in diameter with a flux of ∼9.5 × 10^10^ photons s^−1^ at the peak of the Se *K* absorption edge (λ = 0.979 Å) on beamline ID23-1 at the ESRF. Crystals of this system (20–50 µm in the largest dimension) diffract rather poorly; therefore, in order to increase the data multiplicity to allow a more accurate determination of anomalous differences, six different sample holders were used in this experiment. The initial mesh scans produced heat maps (Fig. 7 ▸ *a*) used to direct the collection of 137 partial data sets, 122 of which could be automatically processed and 45 of which were merged to produce a final data set to *d*_min_ = 3.46 Å after HCA (Table 1 ▸, Figs. 7 ▸ *b* and 7 ▸ *c*). Structure solution (Figs. 7 ▸ *d* and 7 ▸ *e*) was carried out using the SAD technique as implemented in the *CRANK*2 pipeline (Skubák & Pannu, 2013 ▸).

## 4. Discussion   

### 4.1. General comments   

The method that we describe here, while similar to the multi-crystal data-collection methods for samples mounted in micro-meshes described previously (Soares *et al.*, 2014 ▸), presents fundamental differences. Notably, a very low X-ray dose pre-screening of a sample mount is used to both identify the positions of crystals contained on the sample mount and to rank the diffraction characteristics of the crystals in order to create a priority for the subsequent automatic collection of partial data sets, and a HCA protocol is used to choose which partial data sets to merge to produce the best final data set. Moreover, when the sample holder contains a series of crystals much larger than the X-ray beam the method also automates the type of multi-crystal/multi-position data collection (Riekel *et al.*, 2005 ▸) that has become essential in the structural study of G protein-coupled receptors (GPCRs; Rasmussen *et al.*, 2011 ▸; Hollenstein *et al.*, 2013 ▸; Lebon *et al.*, 2011 ▸). Furthermore, for crystals larger than the X-ray beam the rapid online analysis and ranking of diffraction characteristics using *DOZOR* (§[Sec sec2.1]2.1) also provides diffraction cartographs (Bowler *et al.*, 2010 ▸) of the crystals contained on the sample mount, ensuring that partial data sets are only collected from well diffracting areas of any given crystal.

To demonstrate the general applicability of the workflow described here, we have applied it to various systems and scenarios in which many crystals of the same type are mounted on the same cryocooled sample holder. In all of the cases presented our workflow has yielded data sets that are fit for purpose (Table 1 ▸, §[Sec sec4.2]4.2). As might be expected (Fry *et al.*, 1996 ▸), the protocol described here is particularly amenable to systems (*i.e.* thaumatin, bacteriorhodopsin, Maelstrom) that crystallize in high-symmetry space groups. However, our experiments using monoclinic crystals of lysozyme show that the method can also be applied to low-symmetry systems. Furthermore, as the monoclinic form of lysozyme crystallized as clumps of intergrown crystals (Fig. 5 ▸ *a*), the success of this latter experiment demonstrates that the protocol developed also automates the collection of diffraction data using mini-focus or micro-focus X-ray beams under conditions where mounting single crystals of a particular sample may prove to be difficult or impossible.

It is worth noting that the completeness of the data set obtained for monoclinic lysozyme following the HCA-directed merging of the partial data set collected is rather incomplete (21 of 40 automatically processed partial data sets merged, 85% completeness; Table 1 ▸). However, this is not the result of a combination of low-symmetry crystals lying in preferred orientations in the sample holder. Indeed, merging 39 of the 40 automatically processed partial data sets greatly improves the completeness (Fig. 5 ▸ *g*). However, the quality of the resulting data set is seriously degraded compared with that obtained by merging only partial data sets in the main HCA cluster (Fig. 5 ▸ *g*). Moreover, in contrast to what is observed following HCA-directed merging, the resulting difference electron density does not allow the proper identification of nitrate ions bound to the protein (Figs. 5 ▸ *f* and 5 ▸ *h*). It is thus clear that HCA is an indispensable tool for the proper merging of partial data sets. Nevertheless, that the merged data set for monoclinic lysozyme obtained following HCA is somewhat incomplete suggests, for some low-symmetry systems at least, that data collection from samples in two loops with different orientations in the X-ray beam may be required to ensure a fully complete, high-quality data set.

The examples that we present include the compilation of complete diffraction data from partial data sets collected from a series of microcrystals (∼5 µm in the largest dimension), contained on the same sample holder, of a membrane protein (bacteriorhodopsin) grown in lipidic mesophase. Such mesophases are very important media for the growth of membrane-protein crystals (Gordeliy *et al.*, 2003 ▸), but are often opaque in nature, particularly when cooled. It can thus be challenging to identify, mount and centre in the X-ray beam small crystals produced in such media. That the workflow described here uses diffraction-based methods to identify the positions of crystals in a sample holder is clearly a major advantage in such cases as it obviates such problems, particularly when entire crystallization drops are harvested, by automating the collection of partial data sets from multiple crystals.

### 4.2. Structure solution and refinement   

#### 4.2.1. Diffraction data for structure solution by molecular replacement   

The examples of bacteriorhodopsin (BR1 and BR2), thaumatin and monoclinic lysozyme described above clearly show that the protocol that we have developed yields, even for very small crystals, complete diffraction data sets that allow structure solution by MR. Moreover, despite the fact that all data sets were obtained by the merging of multiple partial data sets, electron-density (2*mF*_obs_ − *DF*_calc_, α_calc_) and difference density (*mF*_obs_ − *DF*_calc_, α_calc_) maps calculated during structure refinement clearly allow the identification of moieties not included in the MR search models: retinal (BR1 and BR2; Figs. 2 ▸ *f* and 3 ▸ *f*), tartrate (thaumatin; Fig. 4 ▸ *f*) and NO_3_^−^ (monoclinic lysozyme; Fig. 5 ▸ *f*). This suggests that the method developed may, in the future, have a significant role to play in projects aimed at fragment screening (Murray & Blundell, 2010 ▸) as an aid in drug design. Traditionally, such projects are based around the production of relatively large, robust crystals for use in soaking experiments (Oster *et al.*, 2015 ▸). However, the results presented here show that this clearly does not need to be the case and that complete, high-quality data sets could straightforwardly be compiled from a series of smaller crystals mounted on the same sample holder. Moreover, as evidence suggests that smaller crystals require reduced fragment/ligand-soaking times to obtain the same occupancy of the fragment/ligand in crystal structures (Cole *et al.*, 2014 ▸), microcrystal-based fragment screening experiments may well become the norm, with soaking times based on the largest crystal contained in the crystallization drop ensuring the maximum occupancy of ligands/fragments in all of the crystals mounted on a single sample loop.

#### 4.2.2. Diffraction data for structure solution exploiting anomalous scattering   

In order to demonstrate the possibilities of the workflow presented here to produce data suitable for experimental phasing techniques that exploit anomalous scattering, two different systems were investigated. The first of these, thermolysin, contains one catalytic Zn^2+^ ion and three Ca^2+^ ions per protein chain (316 residues), producing a theoretical anomalous diffraction ratio (〈Δ*F*/*F*〉) of ∼2% for data collected at the peak of the Zn *K* absorption edge. The second, the selenomethionyl derivative of the MAEL domain of *B. mori* Maelstrom (Chen *et al.*, 2015 ▸), produces a theoretical anomalous diffraction ratio of 4.0% for data collected at the peak of the Se *K* absorption edge. However, the crystals of this system diffract rather poorly (see Table 1 ▸). The collection of data of sufficiently high quality for the structure solution of both systems is thus clearly challenging, even from single crystals. Nevertheless, as can be seen in Figs. 6 ▸ and 7 ▸, for both systems our multi-crystal workflow clearly yields diffraction data of sufficient quality for structure solution. As might be expected, a high data multiplicity was important in both cases (Table 1 ▸) and to achieve this for Maelstrom required combining partial data sets from crystals mounted on six different sample holders (Fig. 7 ▸ *a*).

### 4.3. Perspectives   

We have developed an automatic procedure to locate, rank the diffraction characteristics of and collect partial data sets from large numbers of crystals contained on the same sample holder. Subsequent HCA of the partial data sets collected then allows the choice of which partial data sets to merge to produce a final data set for downstream structure solution and refinement. Compared with previously presented SSX protocols (Gati *et al.*, 2014 ▸; Nogly *et al.*, 2015 ▸; Stellato *et al.*, 2014 ▸), *MeshAndCollect* has several advantages, notably that small but contiguous data sets can, if desired, be collected from all crystals contained on the sample holder. Crystal wastage is thus not an issue, data reduction from raw diffraction images to structure factors and standard deviations is comparably straightforward and the quality of the final data set is improved. Moreover, the experiments described in §[Sec sec3]3 clearly demonstrate the capability of *DOZOR* to detect diffraction signal in low-dose two-dimensional mesh scans even for the smallest crystals (BR2; §3.1) studied in this work, which had an average volume of ∼50 µm^3^.

When starting this work, we presumed that cryocooled crystals contained on the same loop would be relatively isomorphous as all crystals are from the same crystallization drop and subject to similar handling during mounting and cryocooling (Giordano *et al.*, 2012 ▸). The dendrograms shown in Figs. 2 ▸, 3 ▸, 4 ▸, 5 ▸ and 6 ▸ suggest that this is the case, although in several of our examples many of the partial data sets collected are not used to construct the final result. Most of the above histograms contain one main cluster with high mutual correlation coefficients and a continuum of data sets with decreasingly low correlation to the main cluster. Such a pattern is indicative of strongly varying data quality between partial data sets rather than crystal non-isomorphism and suggests that some partial data sets were collected from positions with overlapping crystal lattices or other issues such as crystal damage. Clearly, the evaluation of initial two-dimensional mesh scans with *DOZOR* did not filter such positions out. Furthermore, with only a 10° rotation range measured at each position it is difficult to detect such problematic data sets on the basis of their internal processing statistics, and HCA is required to filter out the worst partial data sets. In the case of Maelstrom, where partial data sets were measured from crystals on several different sample mounts, the dendrogram (Fig. 7 ▸) shows well populated clusters above a cutoff of dist(*i*, *j*) = 0.15 and a continuum of poorly correlated data sets below this cutoff. This suggests that both non-isomorphism and variation in data quality between partial data sets is present. However, as can be seen, both poor-quality and non-isomorphous partial data sets are successfully filtered by the HCA procedure.

Despite the success of the experiments described above, the procedure developed will eventually be improved in many areas. Here, all samples were mounted and cryocooled manually, and it may be that better results can be achieved by taking advantage of robotic crystal-handling methods both for the removal of mother liquor from the crystallization drop and the mounting and cryocooling of crystals in a suitable sample holder (Cipriani *et al.*, 2012 ▸). Moreover, for the different experiments described here the total absorbed doses per crystal (Table 1 ▸; calculated post-experiment using *RADDOSE*; Paithankar & Garman, 2010 ▸) are rather low compared with the Henderson/Garman limits (Henderson, 1990 ▸; Owen *et al.*, 2006 ▸) generally used in diffraction data collection from cryocooled single crystals of macromolecules. In future versions of the pipeline presented here, following the low-dose two-dimensional mesh scan the optimum total exposure time per crystal (partial data set) will be calculated before the data-collection step using the *EDNA* characterization software (Bourenkov & Popov, 2010 ▸; Incardona *et al.*, 2009 ▸), the result being better quality and/or higher resolution data collected per crystal. For crystals that are highly radiation-sensitive one might even imagine the use of a ‘Burn Strategy’ workflow (Leal *et al.*, 2011 ▸) to provide a precise estimation of the maximum allowable total absorbed dose per crystal.

As the *EDNA* procedure implies the indexing of diffraction patterns (Incardona *et al.*, 2009 ▸), comparison, for crystals larger than the X-ray beam, of orientation matrices will allow either the pre-clustering of partial data sets collected from different points on the same crystal or the measurement of crystal size and alignment in the sample holder. In the latter case this information could be used to automatically guide helical data collections (Flot *et al.*, 2010 ▸; de Sanctis *et al.*, 2012 ▸) that, provided that diffraction is homogenous, may allow the collection of complete data sets from each of the different crystals contained in the sample holder. For crystals of a similar or smaller size than the X-ray beam prior knowledge of the crystal orientation in the X-ray beam will allow a broader range of experiments than is currently the case. In particular, the order of the collection of partial data sets could be constructed to ensure the compilation of a complete data set when only a few crystals are available or to ensure the collection of as highly redundant data as possible. Finally, for sample mounts containing many small robust, well diffracting crystals one can also imagine a modification to the pipeline in which complete diffraction data sets for structure solution and subsequent refinement are collected from all crystals contained in the sample holder. Separating such data sets into different clusters would result in ensembles of crystal structures for each target.

Once data collection and processing have been completed, a final improvement to the pipeline is in the choice of partial data sets to merge to produce a final data set. This choice clearly depends on the aim of the experiment in hand (*i.e.* structure solution by molecular replacement, *de novo* structure solution using SAD *etc.*), and in principle is best made using HCA based on CC_*I*_(*i*, *j*) (§[Sec sec2]2; Giordano *et al.*, 2012 ▸). However, for partial data sets from low-symmetry crystals the number of common unique reflections for each pair of data sets may be low, thus leading to artefacts, and a better approach may be to combine HCA with the type of ‘scale-and-merge’ algorithms currently implemented in the *PHENIX* package (Adams *et al.*, 2010 ▸; https://www.phenix-online.org/version_docs/dev-1977/reference/scale_and_merge.html) or recently described for other SSX protocols (Huang *et al.*, 2015 ▸).

## 5. Conclusions   

We have presented here a pipeline for the routine collection of partial diffraction data sets from many randomly oriented crystals of the same biological macromolecule contained in a single cryocooled sample holder. The major advantages of the pipeline developed are (i) that it can be applied to crystals mounted in almost any available sample holder suitable for cryocooling, thus rendering the methodology available to the widest possible range of potential users, (ii) that the positions of all well diffracting crystals are determined and that their diffraction strength is ranked prior to data collection, (iii) that small, but contiguous, partial data sets are collected from as many crystals contained in the sample holder as is desired and (iv) that HCA is used to choose partial data sets for merging to produce the best possible data set for downstream analysis and structure solution. As described above, the protocol developed can be applied to both SSX-type experiments involving microcrystals and to multi-position data collection from crystals larger than the X-ray beam size. The results presented here suggest that the method developed will be useful in all areas of macromolecular crystallography, including the compilation of a complete data set from many very small crystals (∼5 µm in the largest dimension), in structure determination exploiting anomalous scattering and in projects aimed at rational drug design.

While we have confined our experiments to crystals mounted on cryocooled sample holders, there is no reason, providing that the increased radiation damage is taken into account, that the automated screening and data-collection procedure developed cannot also be applied at room temperature, particularly in experiments that involve *in situ* screening and data collection (Axford *et al.*, 2012 ▸; Jacquamet *et al.*, 2004 ▸; le Maire *et al.*, 2011 ▸; Huang *et al.*, 2015 ▸). Moreover, *MeshandCollect* should also be extendable to structure solution based on radiation damage-induced phasing (RIP; Ravelli *et al.*, 2003 ▸; de Sanctis & Nanao, 2012 ▸) or SAD experiments based on inverse-beam protocols (González, 2003 ▸).

## Supplementary Material

PDB reference: thermolysin, 5a3y

PDB reference: lysozyme, 5a3z

PDB reference: bacteriorhodopsin, 5a44

PDB reference: 5a45

PDB reference: thaumatin, 5a47

## Figures and Tables

**Table 1:** Data collection and processing Values in parentheses are for the outer shell.

	BR1	BR2	Thaumatin	Lysozyme	Thermolysin	Maelstrom
Diffraction source	ID29, ESRF	ID29, ESRF	ID29, ESRF	ID23-1, ESRF	ID23-1, ESRF	ID23-1, ESRF
Wavelength ()	0.976	0.976	0.969	0.979	1.282	0.979
Temperature (K)	100	100	100	100	100	100
Detector	PILATUS3 6M	PILATUS3 6M	PILATUS3 6M	PILATUS 6M	PILATUS 6M	PILATUS 6M
Beam diameter (m)	20	10	10	10	10	10
Flux (photonss^1^)	3 10^11^	1.5 10^11^	8.7 10^11^	3.5 10^10^	4.0 10^10^	9.5 10^10^
Absolute dose per point, two-dimensional grid (MGy)	0.013	0.16	0.016	0.085	0.17	0.34
Absolute dose per partial data set (MGy)	3.4	6.8	2.1	11.9	20.4	4.5
Partial data sets collected	10	59	100	54	96	137
Partial data sets processed	10	38	78	40	77	122
Partial data sets merged	9	10	74	21	49	45
Space group	*P*6_3_	*P*6_3_	*P*4_1_2_1_2	*P*2_1_	*P*6_1_22	*H*32
Unit-cell parameters (, )	*a* = *b* = 61.13, *c* = 110.31	*a* = *b* = 61.25, *c* = 110.89	*a* = *b* = 57.93, *c* = 150.64	*a* = 27.58, *b* = 62.64, *c* = 59.55, = 91.06	*a* = *b* = 92.87, *c* = 92.87	*a* = *b* = 109.95, *c* = 623.53
Resolution range ()	20.052.29 (2.412.29)	19.732.54 (2.682.54)	19.761.19 (1.261.19)	19.731.59 (1.681.59)	19.881.27 (1.331.27)	20.103.46 (3.653.46)
Total No. of reflections	47395	37209	4188764	96305	3330113	562987
No. of unique reflections	9802	7306	81704	23004	80728	19167
Completeness (%)	92.7 (90.6)	96.7 (81.4)	99.4 (96.1)	85.0 (82.1)	92.0 (53.4)	98.7 (94.6)
Multiplicity	4.8 (4.6)	5.1 (4.3)	51.3 (48.4)	4.2 (4.0)	41.3 (8.6)	29.4 (27.7)
Half-set correlation CC_1/2_	0.998 (0.409)	0.938 (0.263)	0.999 (0.692)	0.989 (0.541)	0.998 (0.321)	0.997 (0.39)
*I*/(*I*)	13.0 (2.7)	4.8 (1.2)	48.7 (2.8)	8.0 (2.2)	25.4 (2.7)	9.5 (1.2)
*R*_p.i.m._	0.050 (0.548)	0.181 (0.797)	0.034 (0.503)	0.080 (0.486)	0.017 (0.344)	0.078 (0.638)
*B* factor, Wilson plot (^2^)	37.0	26.5	9.6	12.5	14.9	88.1

**Table 2:** Structure solution and refinement Values in parentheses are for the outer shell.

	BR1	BR2	Thaumatin	Lysozyme	Thermolysin	Maelstrom
Resolution range ()	20.012.29	19.732.57	20.001.20	20.001.59	20.001.27	n.d.
No. of reflections, working set	9318	6924	77618	21804	76712	n.d.
No. of reflections, test set	480	365	3972	1182	3958	n.d.
Final *R*_cryst_	0.232	0.193	0.133	0.213	0.143	n.d.
Final *R*_free_	0.239	0.218	0.151	0.265	0.166	n.d.
Cruickshank DPI	0.0988	0.3826	0.0311	0.1509	0.0467	n.d.
No. of non-H atoms						
Protein	1612	1677	1640	2014	2448	n.d.
Ion					5	n.d.
Ligand	95	20	10	14	31	n.d.
Water	10		290	181	326	n.d.
Total	1717	1697	1940	2209	2810	n.d.
R.m.s. deviations						
Bonds ()	0.012	0.012	0.028	0.008	0.007	n.d.
Angles ()	1.59	1.46	2.18	1.27	1.32	n.d.
Average *B* factors (^2^)						
Protein	39.2	29.5	13.7	16.9	16.5	n.d.
Ion					16.0	n.d.
Ligand	41.3	22.6	10.9	24.3	35.4	n.d.
Water	42.6		29.4	26.0	28.7	n.d.
Total	39.4	29.1	15.2	17.3	17.5	n.d.
Ramachandran plot						
Most favoured (%)	97.1	98.4	99.0	98.0	96.8	n.d.
Allowed (%)	2.8	1.6	1.0	2.0	3.2	n.d.
PDB code	5a44	5a45	5a47	5a3z	5a3y	

## Footnotes

1 The setup of the goniometers on the ESRF beamlines on which our experiments were performed means that, once its position has been identified, each crystal is moved into the X-ray beam. Here, the movement of the crystal is *via* two motors (sampx and sampy) and the rotation axis of the goniometer is not displaced. This movement ensures the correct vertical position of the crystal but leaves open the possibility that the crystal will be misaligned in the direction of the X-ray beam. A misalignment of 10 µm in this direction will result in a misplacement of less than 1 µm over a ±5° rotation. Thus, provided that the beam is larger than 1 µm in size the crystal will remain in the X-ray beam during the data collection.
